# Research on the Performance of Ultra-High-Content Recycled Asphalt Mixture Based on Fine Separation Technology

**DOI:** 10.3390/ma18174140

**Published:** 2025-09-03

**Authors:** Kai Zhang, Hai Zhou, Wenwen Jiang, Wenqiang Wu, Wenrui Yang, Xiangyang Fan

**Affiliations:** 1 Road Material and Structure Engineering Technology Research Center of Jiangxi Provincial, Jiangxi Communications Investment Maintenance Technology Group Co., Ltd., Nanchang 330200, China; 2 School of Civil and Architectural Engineering, East China University of Technology, Nanchang 330013, China; 3 Jiangxi Transportation Investment Group Nanchang East Management Center, Maqiu Town, High-Tech Zone, Nanchang 330108, China

**Keywords:** fine separation technology, recycled asphalt mixture, ultra-high dosage, road performance, mix optimization

## Abstract

To facilitate the high-value utilization of reclaimed asphalt pavement (RAP), this study investigated the efficacy of fine separation technology as a pre-treatment method. This technology significantly reduced the variability of RAP, controlling the coefficients of variation for asphalt content and aggregate gradation within 5% and 10%, respectively, and minimized false particle content (agglomerates of fines and aged asphalt). Response Surface Methodology (RSM) was employed to optimize the mix design for ultra-high-RAP- content mixtures (50–70%). A predictive regression model was developed to determine the Optimal Binder Content (OBC) based on RAP and rejuvenator dosage. The road performance of the resulting mixtures was comprehensively evaluated. Results showed that the technology markedly enhanced the overall performance of recycled asphalt mixtures. While high-temperature rutting resistance improved with increasing RAP content, low-temperature performance declined. The mixture with 70% RAP failed to meet low-temperature cracking requirements. Consequently, an optimal RAP content of 60% is recommended. Furthermore, the generalized sigmoidal model effectively constructed dynamic modulus master curves, accurately predicting the viscoelastic behavior of these ultra-high-RAP mixtures. This study demonstrates that fine separation is a critical pre-processing step for reliably producing high-quality, sustainable asphalt mixtures with RAP content far exceeding conventional limits.

## 1. Introduction

With the ongoing improvement of the highway network, advancements in the transportation industry, and growing traffic volume, asphalt pavements in many regions worldwide have been increasingly damaged. While road construction is being strengthened, the rehabilitation of existing pavements has gained increasing significance. Given the extensive highway maintenance mileage, a large amount of recycled asphalt mixture (RAP) is generated after milling old asphalt pavements [1,2]. The heat recovery process of RAP not only reuses existing materials, thereby reducing landfill waste, but also decreases waste compared to traditional asphalt production methods [3,4]. In addition, incorporating RAP into asphalt mixtures can reduce energy consumption during production, lower the overall demand for raw aggregates, and promote energy conservation, emission reduction, and effective high-volume reuse, which represent future development trends [5,6,7].

At present, Chinese pavement engineering construction and maintenance require billions of tons of sand and gravel materials annually, among which the consumption of asphalt pavement mixtures alone reaches 500 million tons [8]. The energy consumption and emission problems associated with this have become increasingly significant [9]. Meanwhile, annual generation of reclaimed asphalt pavement (RAP) reaches tens of billions of tons. However, despite its value as a recyclable resource, the comprehensive recycling rate is only about 30% [8]. Most of the RAP is disposed of primarily through piling and landfilling [10]. RAP is a multiphase composite material consisting of aged asphalt, reclaimed aggregates, mineral filler, and admixtures. The phenomenon of particle agglomeration represents one of the most notable characteristics of RAP materials. Traditional crushing and screening methods struggle to address the “black stone” phenomenon in RAP [11]. These clumps hinder the effective blending of new asphalt with aged asphalt, resulting in only partial activation of the aged asphalt [12,13,14]. Excessive variability in these RAPs may cause substantial variations in the gradation of recycled asphalt mixtures, complicate mix design, degrade recycling performance, and impair road functionality.

At present, extensive research has primarily focused on the performance of high-content recycled asphalt mixtures. At present, the road industry mostly downgrades the use of recycled asphalt mixtures, applying RAP from the upper asphalt layer to the middle and lower asphalt layers, and the RAP content is usually less than 50%, making it difficult to achieve in situ ultra-high proportion regeneration with RAP content higher than 50% [15,16]. Since the aged asphalt in RAP becomes increasingly brittle with aging, the mixture is prone to cracking in low-temperature environments. Additionally, poor bonding between the original asphalt and aged asphalt results in inadequate toughness and structural strength in the recycled mixture. Moreover, as RAP content increases, the low-temperature performance of the recycled mixture correspondingly declines [17,18]. Li et al. [19] investigated the fatigue damage evolution of high-RAP thermal cycling asphalt mixtures considering creep effects and examined the impact of temperature and stress levels on fatigue life. Their findings indicated that the fatigue life of the mixtures decreased progressively with increasing RAP content. At present, there are different and even contradictory conclusions regarding the performance research of hot-recycled mixtures. Hao et al. [20] compared the mechanical properties of recycled asphalt mixtures under different mixing methods and found that while the high-temperature rutting resistance was diminished by the pre-mixing of new and aged asphalt, water stability and low-temperature performance were improved. Furthermore, the water stability performance of mixtures deteriorates as RAP content increases, although both high-temperature rutting performance and Marshall stability are significantly enhanced [21,22,23,24]. The main reason for the difference in results is that there are many factors that affect the road performance of recycled asphalt mixtures, especially the RAP properties and their variability with different degrees of aging, RAP dosage, and the design of recycled asphalt mixture mix proportions [25,26]. Due to the multiple effects of these factors, it is currently unclear how RAP affects the performance of the final designed recycled asphalt mixture.

However, as discussed, previous studies have been limited by the high variability and agglomeration issues inherent in traditionally processed RAP, which restricts its dosage and compromises mixture performance. To overcome these limitations, this study introduces and evaluates an advanced pre-treatment method: fine separation and screening technology. The primary objectives of this work are threefold: (1) to quantify the effectiveness of this technology in minimizing RAP variability (in asphalt content and gradation) and reducing false particle content; (2) to utilize Response Surface Methodology (RSM) for optimizing the mix design of ultra-high-content RAP mixtures (50–70%); and (3) to comprehensively evaluate the influence of this technology and RAP dosage on the road performance and mechanical properties of the resulting recycled asphalt mixtures.

The RAP oil–stone separation was accomplished using fine separation technology, which effectively separates aged asphalt from aggregates, ensures the ultra-high proportion of RAP usage, and resolves critical issues limiting RAP dosage while enhancing the road performance of plant-mixed recycled mixtures. Secondly, the mix ratios of various RAP recycled asphalt mixtures were optimized using the response surface methodology to determine the optimal oil–stone ratio and regenerant dosage for recycled asphalt mixtures with varying RAP contents. Finally, the road performance and mechanical properties of recycled asphalt mixtures with different RAP contents were evaluated to assess the impact of fine separation technology on their performance.

## 2. The Influence of Fine Separation Technology on the Performance of RAP

### 2.1. Fine Separation Technology

**Figure 1:**
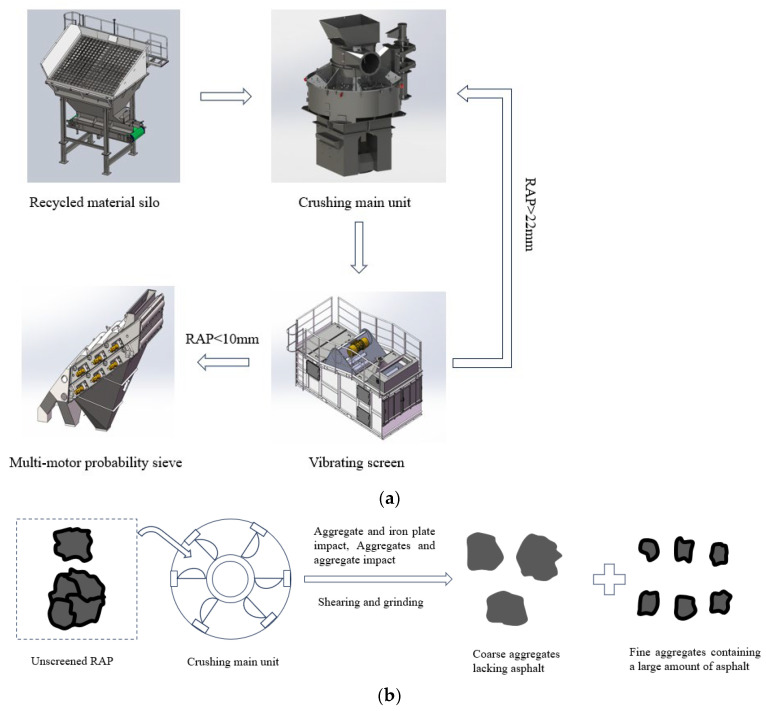
The flow path and principle of the crushing screen: (**a**) the separation process of the crushing screen, (**b**) the principle of crushing.

**Figure 2:**
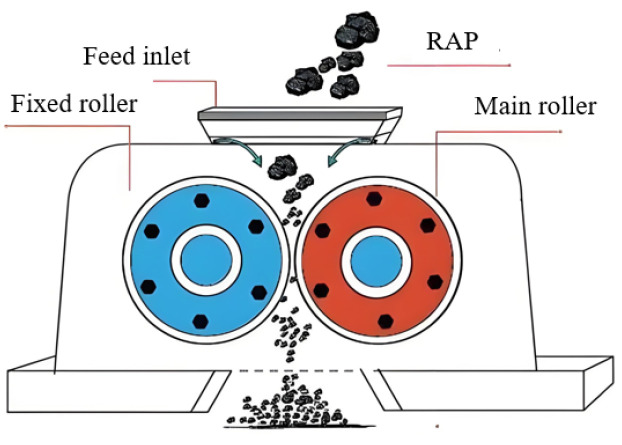
A traditional double-roller crusher, illustrating the crushing mechanism based on compression and shear between counter-rotating rollers. The blue is a fixed roller, and the red is a main roller. The conventional method is less effective at breaking RAP agglomerates compared to the impact-based fine separation process shown in Figure 1.

The RAP material fine separation technology represents an innovative pre-treatment method for aged materials, developed to address the well-documented challenges of variability and agglomeration in traditionally processed RAP [27]. It is capable of dividing RAP into three to five aggregate grades. Before utilizing RAP materials, the technology achieves effective stripping of aged aggregates and facilitates mutual interaction between RAP materials, iron plates, and aged asphalt through rotational abrasion, thereby eliminating agglomeration to the greatest extent and ensuring the ultra-high proportion of RAP usage. The flow path and principle of the crushing screen are shown in Figure 1. Its crushing main unit adopts a vertical shaft impact crusher (Shandong Huaji Heavy Industry Co., Ltd, Weifang, China). After the asphalt aggregate enters the center of the rotor, the high-speed rotating three-port rotor propels the aggregate outward to strike a specially designed anvil for asphalt stripping operations. The impact plate on the iron felt is constructed from specially designed impact-resistant and wear-resistant surfacing plates, ensuring a prolonged service life. The main unit itself has few vulnerable parts, is highly durable, and requires minimal maintenance. By adopting vibrating screens and multi-motor probability screens, the screen mesh can be automatically cleaned, effectively addressing challenges of difficult screening and frequent clogging of asphalt concrete recycled materials. Moreover, the special excitation form of the probability screen achieves optimal screening performance and high efficiency for fine materials. Unlike traditional crushers shown as Figure 2, the fine separation equipment not only separates oil and gravel but also reshapes aggregates, adjusts their geometry, and eliminates irregular particles. This improves the adhesion between aggregates and asphalt, enhancing the overall performance of asphalt mixtures and thereby making it appropriate for high-performance road construction projects.

### 2.2. RAP Performance Analysis and Evaluation

The old asphalt in RAP is integrated into the recycled asphalt mixture, and its content also influences the optimal oil–stone ratio of the recycled mixture. Only by accurately determining the content of old asphalt can the mix ratio design of the recycled mixture be accurately performed. The asphalt content of each RAP grade was determined using the extraction method, and the results are shown in Table 1. As the particle size of the aggregate decreases, the RAP asphalt content increases. The average asphalt content of ≤5 mm particles reached 6.319%, as smaller aggregate particle sizes result in larger specific surface areas, leading to more aged asphalt coating the aggregates. The average asphalt content of the two grades of aggregates, 5–10 mm and 10–22 mm, was 1.988% and 1.718%, respectively, both less than 2%. This was primarily because the fine separation equipment effectively separated oilstone through impact grinding in the crushing main unit, allowing the aged asphalt on the surface of coarse aggregates to be separated and mixed into the fine aggregates. From the perspective of the coefficient of variation, the coefficients of variation for the three grades of aggregates were all less than 5%, indicating that the asphalt content of RAP after fine separation is highly stable, avoiding significant variability across different sampling batches. This consistency ensures that the oil–stone ratio of the recycled asphalt mixture remains consistent and guarantees the consistent performance of the recycled mixture.

The stability of RAP material gradation is a critical factor influencing the properties of RAP aggregates. RAP is inherently characterized by high variability. The instability of RAP gradation often poses challenges in the design of ultra-high-content plant-mixed hot recycling. However, the gradation stability of RAP is significantly improved after fine separation, shown as in Table 2. The coefficient of variation for the gradation of the three aggregate grades follows the pattern (≤5) mm < (5–10) mm < (10–22) mm, with the coefficient of variation increasing progressively as particle size increases. Furthermore, the coefficient of variation in the key sieve holes for all three grades of aggregates remains within 10%, thereby ensuring the gradation stability of the recycled mixture.

### 2.3. Evaluation of RAP False Particle Size

**Figure 3:**
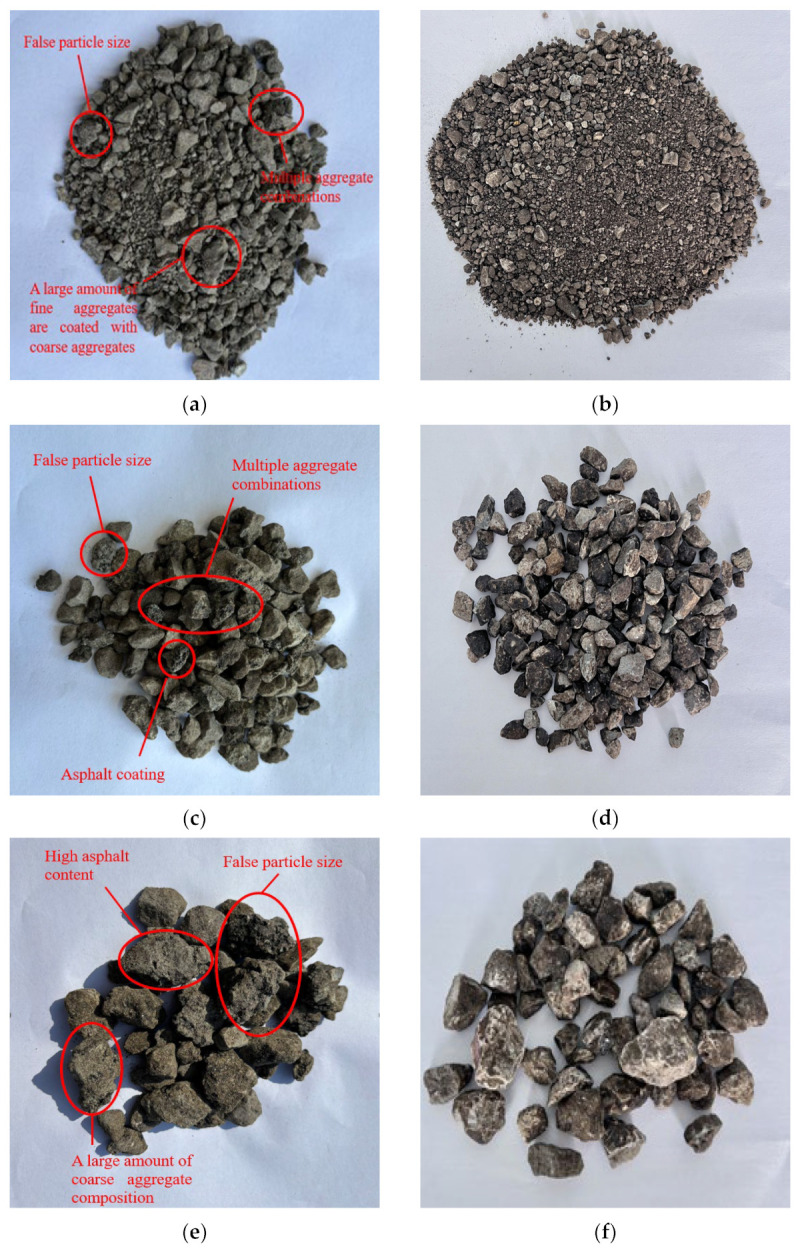
Comparison between traditional crushing and screening and fine separation screening: (**a**) traditional fragmentation ≤8 mm, (**b**) fine separation ≤5 mm, (**c**) traditional fragmentation 8–12 mm, (**d**) fine separation 5–10 mm, (**e**) traditional fragmentation 12–22 mm, (**f**) fine separation 10–22 mm. A visual comparison clearly shows that the fine separation processed RAP (**b**,**d**,**f**) is more liberated and contains fewer agglomerates than the traditionally processed RAP (**a**,**c**,**e**).

Compared with RAP produced by traditional crushing and screening, RAP processed through fine separation offers stable asphalt content, low aggregate gradation variability, and reduced false particle size content, shown as in Table 3. False particle size refers to oversized “RAP clumps” formed by the adhesion of aged asphalt due to incomplete separation of individual RAP particles, which are fully decomposed into separate aggregates after extraction [28,29]. When RAP false particle sizes are not separately mixed and are added as a whole to asphalt mixtures, this leads to segregation in the recycled asphalt mixture. Additionally, these false particle sizes are prone to forming stress concentrations under external forces, resulting in early damage to asphalt pavements. Therefore, false particle sizes not only compromise the gradation stability of recycled asphalt mixtures but also negatively impact their road performance and service life. Figure 3 illustrates the three stages of RAP processed by traditional double-roller crushing and screening, as well as the three stages of RAP processed by fine separation.

It can be known from the test data that the residual screening value of RAP after fine separation is very small. The residual screening value of ≤5 mm is controlled within 5.0%, the maximum residual screening value of 5–10 mm is 7.7%, and the maximum residual screening value of 10–22 mm is 5.2%. It can be seen that the residual screening deviations of the three grades of aggregates before and after pumping are not large. The fine aggregates adhered to the surface of RAP by aged asphalt are relatively few, ensuring the stability of aggregate gradation. After the RAP extraction and the traditional double-roller crushing and screening were compared, the calculated residual screening deviation value of the 4.75 mm screen holes for the ≤8 mm reached 19.2%, and the calculated residual screening deviation value of the 9.5 mm screen holes ranging from 8 to 12 mm was 24.6%. The sieving residue deviation values of the three sieve holes of 13.2 mm, 16 mm, and 19 mm with a diameter of 12–22 mm were all greater than 15%. Moreover, after extraction, the medium and coarse aggregates of 12–22 mm were basically not retained on the sieve, and the false particle size decomposed into individual aggregates, resulting in a deviation value of the 4.75 mm sieve hole as high as −25.6%. The deviation values of the key sieve holes of the three grades of aggregates after traditional double-roller crushing and screening are all too large, making it difficult to ensure the gradation of the recycled mixture after the increase in RAP content, which has a very adverse effect on the performance of the recycled asphalt mixture.

## 3. Mix Optimization of Ultra-High-RAP-Content Asphalt Mixtures Using RSM

When conducting the proportion design of asphalt mixtures, the maximum density curve theory is extensively applied in China [30]. To better investigate the influences of RAP content, regenerant content, and oil–stone ratio on recycled asphalt mixtures, this study examines the middle surface layer recycled asphalt mixture AC-20C (AC represents continuous graded asphalt mixture, while 13 refers to the maximum nominal particle size of the mixture being 13 mm) and conducts proportion optimization using the response surface methodology [31]. The Marshall test investigates recycled asphalt mixtures with varying RAP and regenerant contents that were developed using the response surface methodology. Through RSM software called Design Expert, functional relationships between RAP content, regenerant content, oil–stone ratio, and various physical performance indicators of the mixtures were established. The optimal oil–stone ratios for five different RAP dosages were calculated using the regression equation, and the optimal regenerant dosage was identified.

### 3.1. RAP Gradation Composition Design

With the increase in RAP content, the performance of recycled asphalt mixtures tends to deteriorate. In this study, the middle- and upper-layer AC-20C was selected for mix proportion design. To evaluate the performance of asphalt mixtures with varying RAP contents under the fine separation technology, RAP contents of 30%, 40%, 50%, 60%, and 70% were selected for mix proportion design. The mineral synthesis gradation of the recycled asphalt mixture is presented in Table 4.

### 3.2. Design of Response Surface Method Scheme

Through the Marshall test of the middle- and surface-layer-modified plant-mixed hot-recycled asphalt mixture AC-20C with varying RAP dosages and regenerant dosages (shown in Table 5) designed using the central composite method, this study selected RAP dosages (30%, 40%, 50%, 60%, 70%), regenerant dosages (accounting for 1%, 2%, 3%, 4%, 5% of the aged asphalt), and a total oil–stone ratio of 3.5%. The three influencing factors include RAP dosage, regenerant dosage, and total oil–stone ratio, each with levels of 4.0%, 4.5%, 5.0%, and 5.5%. Five levels were selected for each influencing factor. A three-factor and five-level design scheme was employed. The design scheme for the influencing factor levels is presented in Table 6. The Marshall experimental design was conducted for three independent factors and five levels, as shown in Table 7.

### 3.3. Establishment and Analysis of Multiple Regression Equations

**Figure 4:**
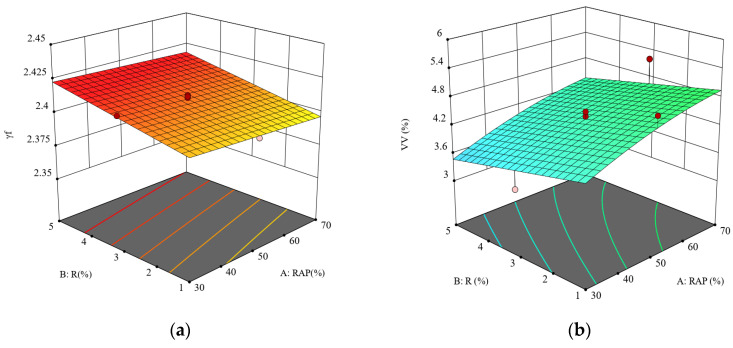

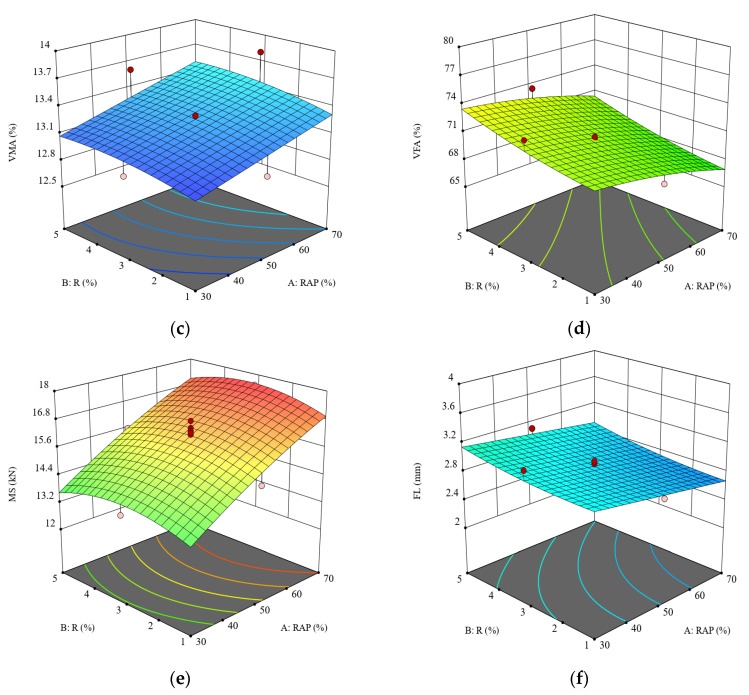
Mechanical property response surface: (**a**) bulk specific gravity of asphalt mixtures, (**b**) percent air voids volume, (**c**) percent voids in mineral aggregate, (**d**) percent voids in mineral aggregate that are filled with asphalt, (**e**) Marshall stability, and (**f**) flow value.

The test data were input into the Design Expert software for fitting analysis, and the response surfaces of quadratic polynomial models for each physical performance index, derived using the response surface methodology, are presented in Figure 4.

**Figure 5:**
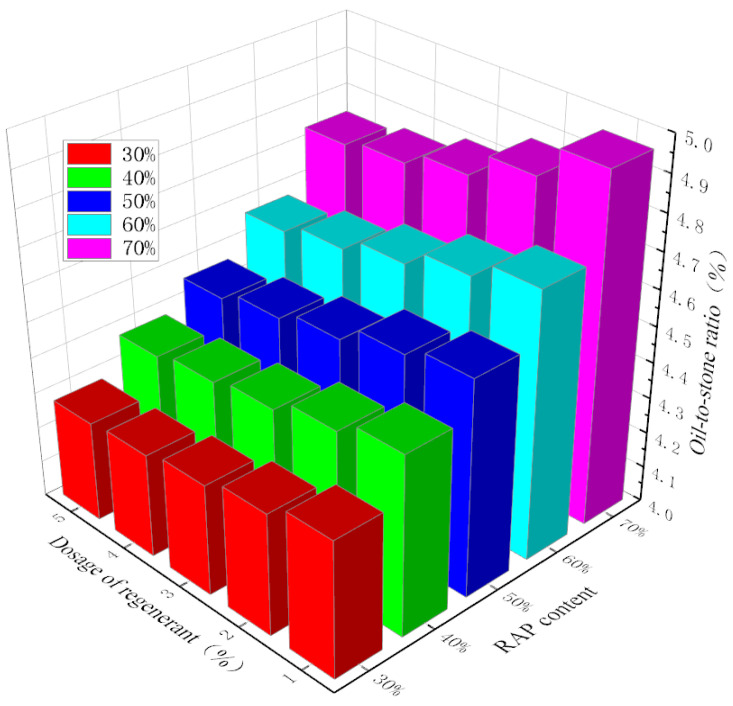
The oil–stone ratio under different RAP dosages and regenerant dosages.

Based on the results of the central composite design test and the Marshall test, multiple quadratic regression equations developed using Design Expert software were used to determine the optimal oil–stone ratio of the hot-recycled modified asphalt mixture under various RAP and regenerant dosages. The results are shown in Figure 5. Among them, a_1_, a_2_, a_3_, and a_4_ represent the maximum relative density of the gross volume, maximum stability, median void ratio, and median asphalt saturation of the recycled asphalt mixture, respectively. OAC_1_ is the average value of a_1_ to a_4_. OAC_min_ and OAC_max_ are the minimum and maximum values of the oil–stone ratio required to meet the performance indicators specified by the standards. OAC_2_ is the average value of OAC_min_ and OAC_max_, and OAC, the optimal oil–stone ratio for recycled asphalt mixtures, is the average value of OAC_1_ and OAC_2_.

With the increase in RAP content, the optimal oil-to-aggregate ratio shows a rising trend. This is mainly due to the accumulation of aged asphalt increasing the optimal asphalt content. Because this aged asphalt is very hard and has limited availability, additional new asphalt is needed to ensure appropriate aggregate asphalt film thickness, asphalt mixture workability, and long-term road performance [32]. In addition, a small amount of regenerant is insufficient to fully activate all the aged asphalt, necessitating the addition of new asphalt to enhance the performance of the recycled asphalt mixture [33]. Taking the 70% RAP content as an example, when the regenerant content increases from 1% to 5%, the optimal oil-to-aggregate ratio gradually decreases by 1.4%, 2.6%, 3.6%, and 4.5%. By increasing the regenerant content, the requirement for new asphalt can be effectively reduced. That is, the regenerant can partially replace new asphalt and reduce overall asphalt consumption. However, regenerants are expensive. To achieve large-scale production of factory-mixed hot-recycled modified asphalt mixtures in the future, it is necessary to reasonably select the regenerant dosage. When the RAP content is 70%, with the increase in regenerant content, the differences in the optimal oil-to-aggregate ratio are 0.09%, 0.07%, 0.04%, and 0.02%. When the regenerant content is 1% and 2%, the differences in the optimal oil-to-aggregate ratio are too large. However, when the regenerant content reaches 4% and 5%, its addition does not significantly decrease the requirement for new asphalt. Therefore, in this study, 3% regenerant was selected as the optimal dosage to conduct performance studies on five hot-recycled modified asphalt mixtures AC-20C with different RAP dosages.

## 4. Research on the Performance of Ultra-High-Content Factory-Mixed Hot-Recycled Asphalt Mixture

### 4.1. Water Stability Performance

#### 4.1.1. Immersion Marshall Test

**Figure 6:**
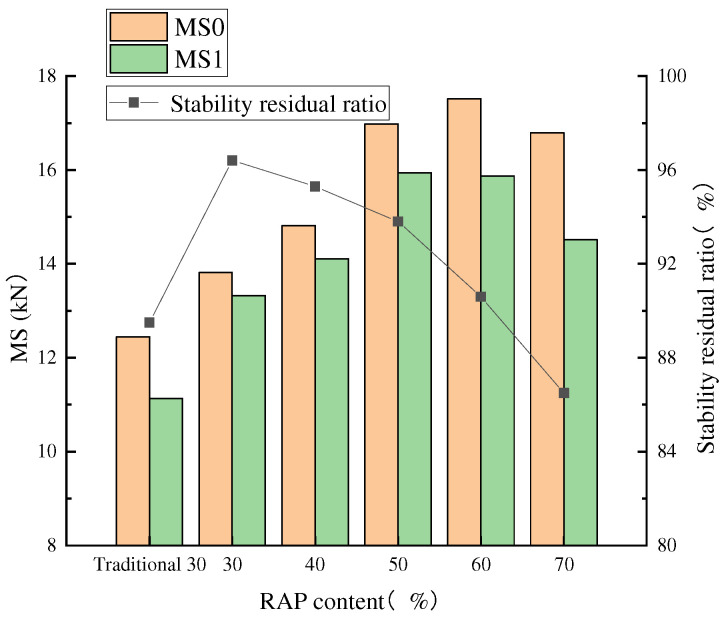
Residual ratio of Marshall stability.

The water immersion Marshall test involves curing one group of specimens in a constant-temperature water tank at 60 °C for 30 min and then measuring their stability (MS), while another group is cured under the same conditions for 48 h, and then their stability (MS1) and flow values are measured. The residual ratio (MS0) is used as the water stability evaluation index for asphalt mixtures [34]. The water stability performance test results of RAP recycled asphalt mixtures with different dosages, obtained from the immersion Marshall test, are presented in Figure 6.

Figure 6 indicates that the stability of the recycled asphalt mixture processed using traditional crushing and screening at a 30% rate is lower compared to the recycled asphalt mixture processed using fine separation at the same rate. Due to the poor separation effect of traditional double-roller screening methods for oilstone, the content of false particle size is relatively high. The new asphalt and regenerant fail to fully blend with the aged asphalt. The aged asphalt encapsulates the old aggregates, resulting in clumping. The mixture exhibits poor bonding performance with the new aggregates, while uneven mixing further contributes to a relatively low residual stability.

Figure 6 provides a direct performance comparison that underscores the efficacy of the fine separation process. The mixture containing 30% traditionally processed RAP exhibits lower stability than its fine-separation-processed RAP counterpart. This is a critical finding, as it quantitatively demonstrates that the superior separation efficiency of the advanced process—specifically its ability to minimize RAP agglomerates and ensure a more homogeneous blend—translates directly into enhanced mechanical properties, even at a moderate RAP dosage. This foundational improvement is what enables the reliable use of much higher RAP contents, which are explored next.

A consistent trend was observed across all mixtures: as the RAP dosage increased, Marshall stability rose while the residual stability ratio declined, with a more pronounced reduction beyond 50% of RAP. This is primarily due to the increased proportion of aged, less adhesive asphalt, which facilitates water intrusion at the aggregate interface and compromises moisture resistance. Despite this trend, the residual stability for all mixes incorporating fine-stripped RAP remained above 80%, meeting specification requirements.

#### 4.1.2. Freeze–Thaw Splitting Test

**Figure 7:**
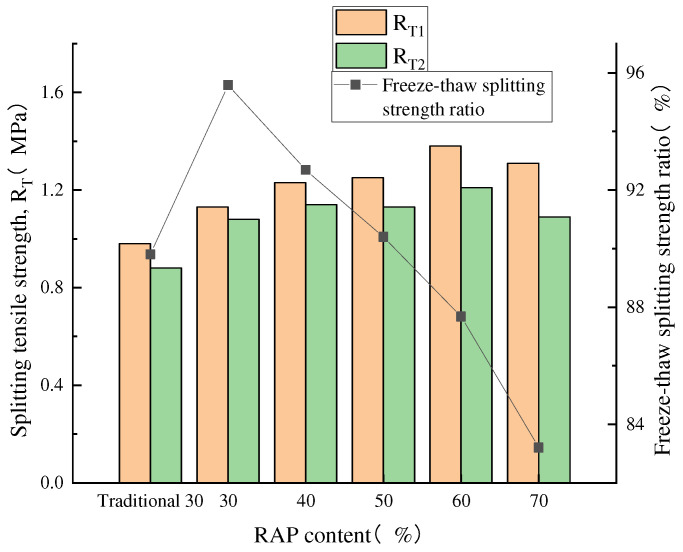
Freeze–thaw splitting strength ratio.

In the freeze–thaw splitting test, the tensile strength ratio (TSR), defined as the ratio of the splitting tensile strength of the specimen after freeze–thaw cycles to that before freeze–thaw, is used as an index to evaluate the water stability performance of the mixture [35]. Figure 7 presents the freeze–thaw splitting test results.

The test data indicate that the freeze–thaw splitting strength ratio of the traditional crushing and screening RAP30% recycled asphalt mixture is 5.78% lower compared to the fine separation RAP30% recycled asphalt mixture. This is primarily attributed to the presence of false particle sizes, which result in uneven mixing of the recycled asphalt mixture and an increased number of weak surfaces. After the Marshall specimens are vacuum saturated with water, water films are more likely to form on the weak surfaces of the mixture, thereby reducing its adhesion.

The freeze–thaw splitting test results revealed a dual effect of increasing RAP content. While the splitting tensile strength steadily increased due to the enhanced hardness imparted by the aged asphalt, the freeze–thaw splitting strength ratio (TSR) exhibited a progressive decline. This decrease in water resistance accelerated beyond 50% RAP, with the 70% RAP mix showing a 12.37% lower TSR than the 30% mix. This is attributed to the embrittlement of aged asphalt, which creates a weaker bonding interface more susceptible to damage from freeze–thaw stresses and water infiltration. Despite this decline, the TSR for all mixtures processed with fine separation met specification requirements, confirming the technology’s effectiveness. These findings are consistent with the immersion Marshall test, collectively demonstrating a trade-off between strength and moisture resistance as RAP content rises.

### 4.2. High-Temperature Performance

**Figure 8:**
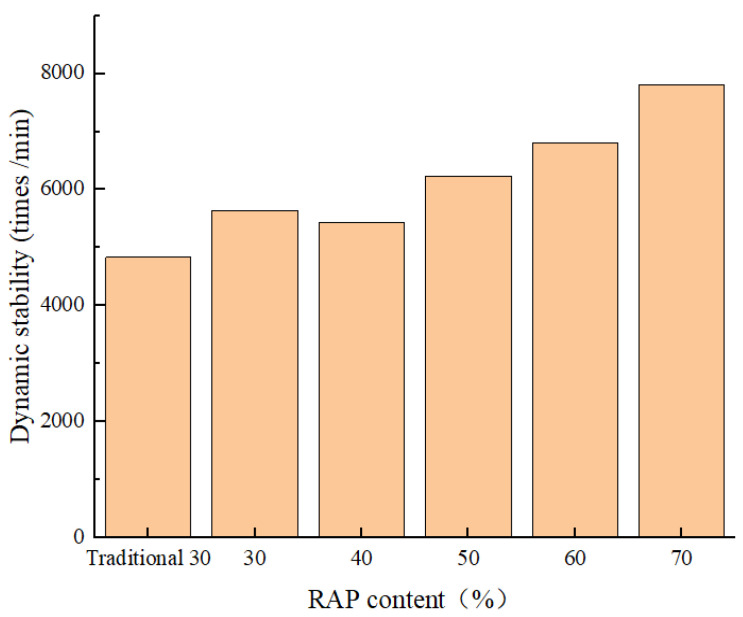
The relationship between RAP content and dynamic stability.

When the temperature is high, the internal bonding material of the asphalt mixture softens under the influence of high temperatures. During vehicle operation, rutting occurs on the asphalt road surface, adversely impacting driving comfort and safety. The high-temperature performance of the fine separation factory-mixed hot-recycled asphalt mixture was evaluated through indoor high-temperature rutting tests [34]. The experimental results are presented in Figure 8.

The fine separation technology markedly improved high-temperature performance. The dynamic stability of the 30% RAP mixture produced with this method was 810 times/mm higher than that of a mixture using traditionally processed RAP. This is a direct result of the technology’s ability to reduce false particles and ensure a coarser, more optimal gradation with sufficient coarse aggregates. Furthermore, a strong correlation was observed between RAP content and rutting resistance: dynamic stability increased by 38.2% as the RAP content rose from 30% to 70%. This consistent improvement is attributed to the hardened nature of the aged asphalt within RAP. The increased viscosity and reduced flexibility of this binder cause the overall mixture to stiffen, thereby significantly enhancing its resistance to permanent deformation under high temperatures and loading.

### 4.3. Low-Temperature Performance

**Figure 9:**
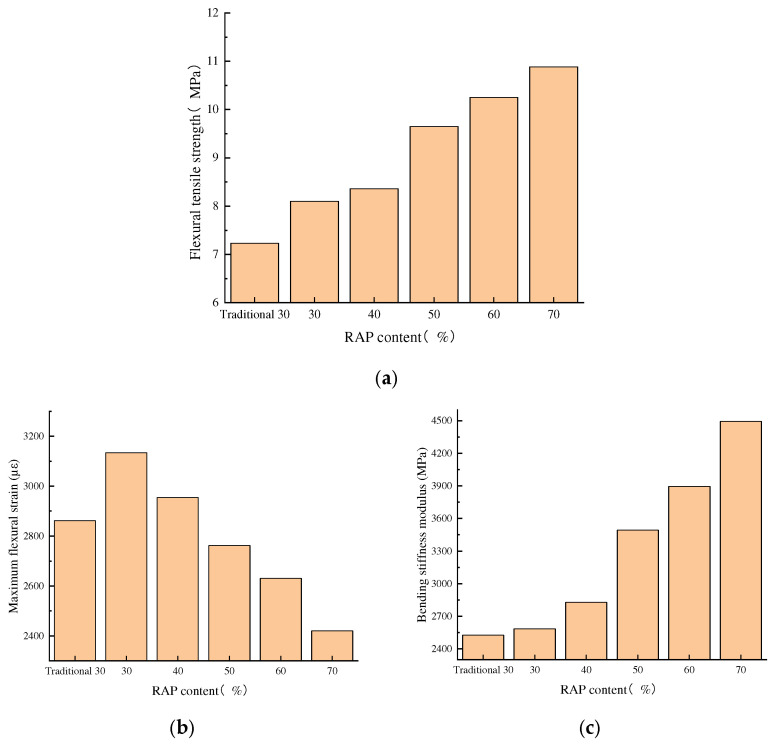
Test results of low-temperature trabeculae with different RAP dosages: (**a**) flexural tensile strength, (**b**) maximum flexural strain, and (**c**) bending stiffness modulus.

The low-temperature performance of asphalt mixtures refers to their ability to resist the formation of shrinkage cracks under low-temperature conditions. Due to the excessive content of old asphalt, the material hardens as a result of prolonged aging during service, and its low-temperature toughness gradually deteriorates, leading to reduced low-temperature performance in recycled asphalt mixtures. Under the influence of external loads and temperature changes during actual service, the mixture becomes more susceptible to deformation and cracking. The low-temperature performance of the recycled asphalt mixture was evaluated through the low-temperature girder test [34], with the experimental results presented in Figure 9.

The low-temperature performance of recycled asphalt mixtures can be partially represented by the low-temperature failure strain of small beam bending. The flexural tensile strength, flexural stiffness modulus, and maximum flexural tensile strain of the mixture with 30% RAP content processed through fine separation are all higher than those achieved through traditional crushing and screening, primarily due to the superior separation efficiency of old asphalt and aggregates achieved through fine separation. Grinding and impacting the old asphalt into fine powder facilitates better blending of new asphalt with old asphalt. Meanwhile, reducing false particle size ensures more uniform mixing of the mixture, thereby reducing segregation in the recycled asphalt mixture.

The low-temperature performance tests revealed a critical trade-off. While increasing the RAP content enhanced the flexural strength and stiffness of the mixture, it concurrently reduced the maximum flexural tensile strain—a key indicator of deformation resistance and crack tolerance. This embrittlement is a direct result of the rising proportion of aged, hardened asphalt in the total binder. Most significantly, the mixture with 70% RAP exhibited a maximum strain below the specification requirement of 2500 µε, indicating unsatisfactory low-temperature crack resistance [35]. This failure suggests that at ultra-high dosages, the regenerant could not fully restore the rheological properties of all the aged asphalt, leaving portions of it brittle and ineffective. Consequently, this study identifies 70% as the practical upper limit for RAP content when low-temperature performance is a design constraint. For mixes approaching this limit, increasing regenerant dosage may be necessary to ensure adequate binder rejuvenation and meet performance specifications.

### 4.4. Fatigue Performance

**Figure 10:**
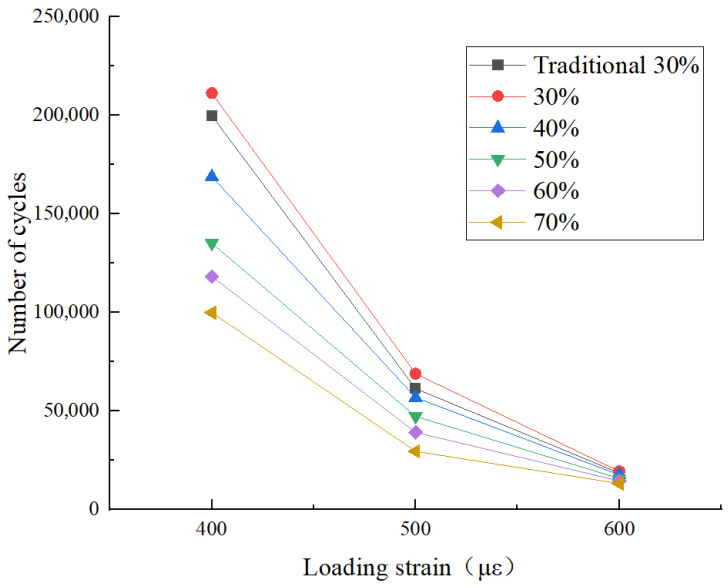
Fatigue test results.

The four-point bending girder test was adopted. The test temperature was 15 °C. The test was conducted at three strain levels of 400 με, 500 με, and 600 με using the controlled strain method [34]. The semi-sinusoidal waveform loading closely simulated the strain loading of vehicle loads on asphalt pavement. The test loading program defined the initial stiffness modulus as the stiffness modulus of the specimen at the 50th loading cycle. When the stiffness modulus of the specimen drops to 50% of the initial stiffness modulus, this indicates significant damage accumulation in the specimen, marking the transition from the stable stage to the failure stage. The test results are presented in Figure 10.

Fatigue life was influenced by two primary factors: applied strain and RAP content. As expected, higher loading strains resulted in shorter fatigue lives for all mixtures due to accelerated crack initiation and stiffness reduction. More critically, the study found that at any given strain level, fatigue life decreased as RAP content increased. This negative correlation is attributed to the increased proportion of aged asphalt, which reduces the overall blending efficiency with virgin binder and creates more weak interfacial zones prone to micro-cracking. Importantly, mixtures produced with fine separation consistently demonstrated superior fatigue performance compared to those using traditional RAP processing. This confirms that the technology’s ability to minimize false particles and improve asphalt-aggregate separation directly enhances the durability and fatigue resistance of the final product.

### 4.5. Mechanical Properties

#### 4.5.1. Analysis of Dynamic Modulus Test Results

**Figure 11:**
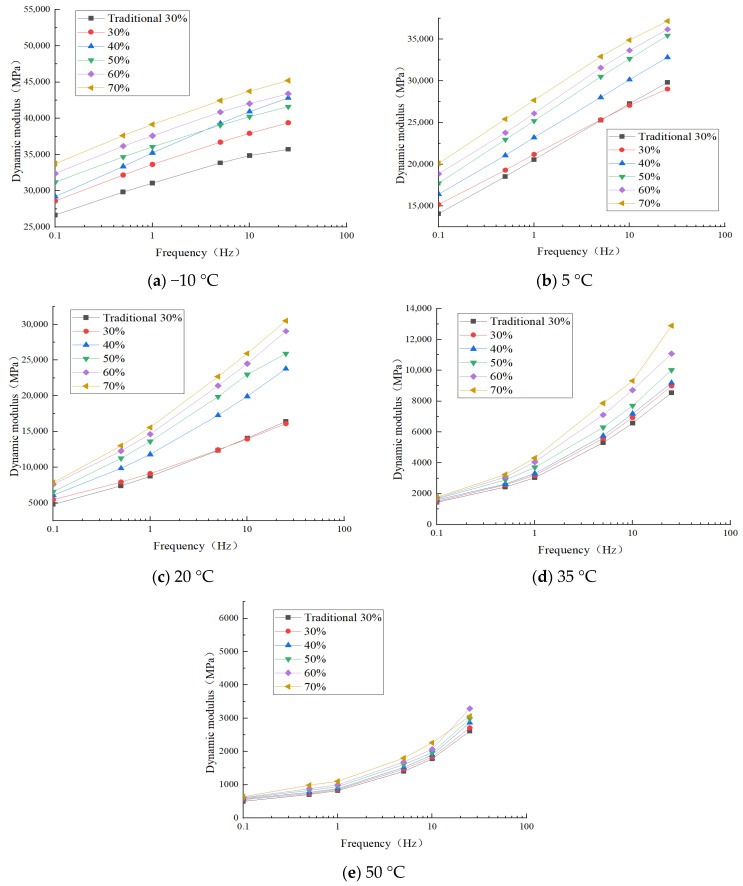
The influence of frequency on dynamic modulus at different temperatures.

Uniaxial compression dynamic modulus tests were conducted to evaluate the properties of the fine separation recycled asphalt mixture. The tests were performed at five temperatures: −10 °C, 5 °C, 20 °C, 35 °C, and 50 °C, with loading frequencies of 25 Hz, 10 Hz, 5 Hz, 1 Hz, 0.5 Hz, and 0.1 Hz [34]. The results of the dynamic modulus test are presented in Figure 11.

Taking a loading frequency of 25 Hz as an example, at −10 °C, the dynamic modulus of the fine separation screening RAP30% is higher than that of traditional screening RAP30%. The primary reason is that under low-temperature and high-frequency conditions, the dynamic modulus of the recycled asphalt mixture is primarily influenced by the mineral material gradation, with the elastic properties of coarse-graded recycled asphalt mixtures being more pronounced. In traditional RAP screening, the higher content of false particle sizes results in a finer overall gradation design, reducing the content of coarse aggregates and consequently leading to lower strength and dynamic modulus. As the test temperature increased from −10 °C to 50 °C, the dynamic modulus of the recycled asphalt mixture with all RAP contents gradually decreased. This can be attributed to the addition of regenerants, which restore the performance of aged asphalt. As a result, the temperature sensitivity of the recycled asphalt mixture approaches that of fresh asphalt mixtures. Under high-frequency loading conditions, the dynamic modulus of the recycled asphalt mixture remains relatively stable regardless of RAP content, indicating that the temperature sensitivity of asphalt pavement is not significantly affected by RAP content. When the temperature approaches 50 °C, the dynamic modulus values of recycled asphalt mixtures with various RAP dosages exhibit minimal differences. This indicates that under high-temperature conditions, the deformation behavior of recycled asphalt mixtures shows reduced differences.

The effect of RAP content on dynamic modulus was temperature-dependent, illustrating a critical performance trade-off. At high temperatures, the dynamic modulus exhibited a clear upward trend with increasing RAP content. This is directly attributed to the rising proportion of hardened, aged asphalt, which increases the overall mixture stiffness and cohesion. This mechanistic stiffening effect correlates strongly with the enhanced rutting resistance observed in earlier tests. Conversely, at low temperatures (e.g., −10°C), this increased stiffness becomes a detriment. The same aged asphalt, with its volatilized light components and oxidized heavy fractions, exhibits significantly reduced viscoelasticity and ductility. Furthermore, the stiff, aged-asphalt film coating the RAP aggregates further diminishes the mixture’s ability to relax stresses. Consequently, the increased dynamic modulus under these conditions is synonymous with a reduction in low-temperature crack resistance.

In summary, the added RAP content simultaneously improves high-temperature performance while compromising the mixture’s low-temperature flexibility.

#### 4.5.2. Dynamic Modulus Master Curve

To investigate the variation in dynamic modulus under different temperatures and loading frequencies, the master curve of the dynamic modulus of recycled asphalt mixtures was established using the time–temperature equivalence principle [36]. This curve was derived from the mechanical properties measured at different test temperatures and loading frequencies [36,37]. The principal curve, established through the generalized sigmoidal model (1) [38], makes it possible to estimate the dynamic modulus at high temperatures and high frequencies, which are challenging to achieve experimentally. This provides additional insights into the mechanical properties of recycled asphalt mixtures processed through fine separation.

(1) $$log\left|E^{*}(f)\right|=d+\frac{a}{{\left[1+le^{b+glog(fa_{T})}\right]}^{\frac{1}{l}}}$$

$E^{*}$—Dynamic modulus of asphalt mixture;

*δ*—Minimum value of dynamic modulus (MPa);

*α*—Difference between maximum and minimum dynamic modulus values (MPa);

*λ*, *β*, *γ*—Shape parameter of dynamic modulus master curve;

*α_T_*—Dynamic modulus master curve displacement factor.

**Figure 12:**
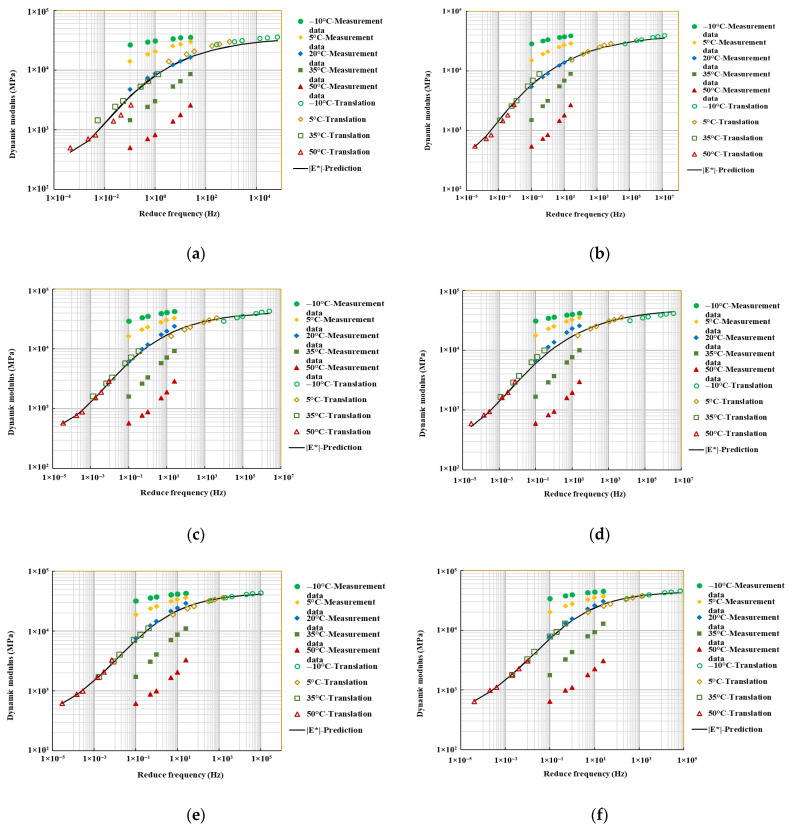
Dynamic modulus master curve: (**a**) Traditional RAP30%, (**b**) RAP30%, (**c**) RAP40%, (**d**) RAP50%, (**e**) RAP60%, (**f**) RAP70%.

As shown in Figure 12, the dynamic modulus of the recycled asphalt mixture significantly increases with rising loading frequency for all RAP dosages. Compared to the traditional screening RAP30% content recycled asphalt mixture, the dynamic modulus of the fine separation RAP30% content recycled asphalt mixture is higher across all frequencies, particularly in the medium- and high-frequency ranges. The reduction in dynamic modulus under high-temperature conditions is mitigated compared to the traditional mixture, indicating that the fine separation technology enhances the high-temperature performance of the RAP mixture.

**Figure 13:**
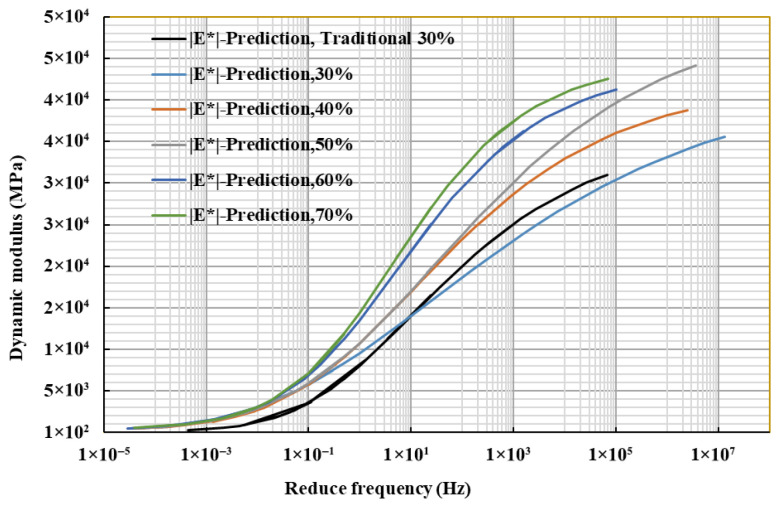
Dynamic modulus corresponding to different RAP dosages.

As shown in Figure 13, the dynamic modulus of the recycled asphalt mixture increased with RAP content, although the rate of this increase gradually diminished. This trend was most pronounced in the medium- to low-frequency ranges (corresponding to high-temperature conditions), where the master curve exhibited a steeper slope and a more significant reduction in modulus. This behavior is attributed to the combined effect of the aged constituents. The RAP aggregates, hardened and textured from years of service, contribute to higher interlocking and frictional forces, increasing overall mixture stiffness. Concurrently, the aged asphalt binder, with its volatilized light components and increased viscosity, provides greater resistance to deformation under load. This synergy between stiffened aggregates and hardened binder amplifies the dynamic modulus, particularly under high-temperature, slow-loading conditions that emphasize the viscous properties of the material.

## 5. Conclusions

### 5.1. Conclusions

To address the challenges of high variability and false particle content in RAP, which hinder its large-scale, high-value utilization, this study investigated the application of fine separation technology for RAP pre-treatment. The primary objectives were to minimize RAP variability, optimize the mix design for ultra-high-content RAP mixtures (30–70%) using RSM, and comprehensively evaluate the resulting performance. The following key conclusions were drawn:

(1) Pre-treatment Efficacy: Fine separation technology successfully mitigated the key challenges of RAP variability, reducing the coefficients of variation for asphalt content and gradation to below 5% and 10%, respectively. This process is essential for ensuring consistent quality in high-RAP mixtures.

(2) Optimized Mix Design: The RSM-based model proved reliable for optimizing the oil–stone ratio across a wide range of RAP (30–70%) and regenerant (1–5%) dosages, providing a practical tool for mix design.

(3) Performance Trade-off: The technology significantly improved mixture uniformity and blending, enhancing overall road performance. A clear performance trade-off was observed: high-temperature stability improved with RAP content, but low-temperature performance and fatigue resistance gradually declined. All performance metrics for the 60% RAP mixture met specifications; however, the 70% RAP mixture failed low-temperature requirements. Therefore, 60% is identified as the optimal and practical maximum RAP content for balanced performance.

(4) Predictive Modeling: The generalized sigmoidal model accurately characterized the viscoelastic properties and constructed master curves, confirming its utility for modeling high-RAP mixtures.

(5) Economic and Environmental Benefits: The ability to reliably incorporate 60% RAP represents a significant economic advantage by drastically reducing the demand for virgin aggregates and asphalt binder. Furthermore, this high-rate recycling offers substantial environmental benefits, including reduced landfill waste, lower energy consumption associated with quarrying and binder production, and a corresponding decrease in the carbon footprint of pavement construction and rehabilitation projects.

### 5.2. Limitations and Future Work

This article successfully proves that fine peeling and screening technology is not only a process but also a necessary condition for achieving high-quality RAP. It can technically achieve a RAP content of 60% and is the best choice for asphalt surface layer. By using RSM, a strong scientific basis can be provided for engineering practice, and high-content RAP asphalt mixtures can be reliably designed based on specific material characteristics and performance goals.

However, in terms of limitations, the long-term aging behavior of ultra-high-RAP mixtures (especially with a 70% content), particularly the possibility of further embrittlement within 5–10 years, requires further research through accelerated aging tests or long-term pavement performance monitoring. Meanwhile, research data shows that a 70% RAP content significantly reduces the water stability and low-temperature performance of asphalt mixtures, making it unsuitable for use in the upper layer of asphalt.

## Figures and Tables

**Table 1:** The content of asphalt content in each grade of RAP after fine separation.

Sampling Number	≤5 mm	5–10 mm	10–22 mm
Average value of asphalt content (%)	6.319	1.988	1.718
Standard deviation	0.229	0.089	0.038
Coefficient of variation (%)	3.6	4.5	2.2

**Table 2:** The passing percentage and variability of RAP in each grade after fine separation.

Materials	Key Sieve Holes (mm)	Average Value	Standard Deviation	Coefficient of Variation (%)
≤5 mm	0.075	17.6	0.38	10.6
	0.6	41.8	0.37	2.2
	1.18	58.8	0.52	3.4
	2.36	73.8	0.43	2.0
5–10 mm	4.75	23.1	2.66	3.5
10–22 mm	9.5	26.0	1.85	4.5

**Table 3:** The false particle size content of RAP in each grade.

Screening Method	Materials	Key Sieve Holes (mm)	Calculate the Residual Deviation of the Sieve (%)
Traditional double-roller crushing and screening	≤8 mm	0.075	−1.4
		1.18	3.6
		2.36	−5.7
		4.75	19.2
	8–12 mm	9.5	24.6
	12–22 mm	13.2	16.5
Fine separation	≤5 mm	0.075	−3.0
		0.6	2.6
		1.18	5.0
		2.36	3.1
	5–10 mm	4.75	7.7
	10–22 mm	9.5	2.9

**Table 4:** Synthetic gradation of AC-20C recycled asphalt mixture.

Sieve Hole Size (mm)	Gradation Type						
	30%	40%	50%	60%	70%	Upper Limit	Lower Limit
26.5	100.0	100.0	100.0	100.0	100.0	100	100
19	93.7	94.1	94.2	94.2	94.2	100	90
16	84.0	85.0	85.3	85.3	85.3	92	78
13.2	70.8	71.1	71.4	71.4	71.4	80	62
9.5	60.6	59.2	59.3	59.3	59.3	72	50
4.75	29.9	29.8	30.2	30.6	30.2	56	26
2.36	22.1	22.2	22.3	22.3	21.7	44	16
1.18	16.2	16.3	16.5	16.7	16.3	33	12
0.6	12.7	12.9	13.1	13.3	13.1	24	8
0.3	9.1	9.2	9.4	9.6	9.5	17	5
0.15	6.8	6.9	7.1	7.3	7.3	13	4
0.075	5.0	5.1	5.3	5.5	5.6	7	3

**Table 5:** Basic performance of petroleum-based regenerant.

Performance Indicators	Unit	Test Results	Specification Requirements [16]
Viscosity (60 °C)	mm^2^/s	121	50–175
Density (25 °C)	g/cm^3^	0.973	measured
Flash point	°C	256	≥220
Appearance	/	reddish brown	measured
Saturation content	%	28.4	≤30
Aroma content	%	32.1	≥30

**Table 6:** Design of influencing factor levels.

Horizontal Design	RAP Content P_RAP_ (%)	Dosage of Regenerant P_R_ (%)	Oil–Stone Ratio P_a_ (%)
−α	30	1	3.5
−1	30	1	3.5
0	50	3	4.5
1	70	5	5.5
α	70	5	5.5

**Table 7:** Central combination design test scheme.

Number	RAP Content PRAP (%)	Dosage of Regenerant P_R_ (%)	Oil–Stone Ratio P_a_ (%)
1	30	1	3.5
2	70	1	3.5
3	30	5	3.5
4	70	5	3.5
5	30	1	5.5
6	70	1	5.5
7	30	5	5.5
8	70	5	5.5
9	30	3	4.5
10	70	3	4.5
11	50	1	4.5
12	50	5	4.5
13	50	3	3.5
14	50	3	5.5
15	50	3	4.5
16	50	3	4.5
17	50	3	4.5
18	50	3	4.5
19	50	3	4.5
20	50	3	4.5

## Data Availability

The original contributions presented in this study are included in the article material. Further inquiries can be directed to the corresponding author.
